# Perinatal compromise affects development, form, and function of the hippocampus part one; clinical studies

**DOI:** 10.1038/s41390-024-03105-7

**Published:** 2024-03-22

**Authors:** Tegan A. White, Suzanne L. Miller, Amy E. Sutherland, Beth J. Allison, Emily J. Camm

**Affiliations:** 1 The Ritchie Centre, Hudson Institute of Medical Research, Clayton, VIC Australia; 2 Department of Obstetrics and Gynaecology, Monash University, Clayton, VIC Australia

## Abstract

**Abstract:**

The hippocampus is a neuron-rich specialised brain structure that plays a central role in the regulation of emotions, learning and memory, cognition, spatial navigation, and motivational processes. In human fetal development, hippocampal neurogenesis is principally complete by mid-gestation, with subsequent maturation comprising dendritogenesis and synaptogenesis in the third trimester of pregnancy and infancy. Dendritogenesis and synaptogenesis underpin connectivity. Hippocampal development is exquisitely sensitive to perturbations during pregnancy and at birth. Clinical investigations demonstrate that preterm birth, fetal growth restriction (FGR), and acute hypoxic-ischaemic encephalopathy (HIE) are common perinatal complications that alter hippocampal development. In turn, deficits in hippocampal development and structure mediate a range of neurodevelopmental disorders, including cognitive and learning problems, autism, and Attention-Deficit/Hyperactivity Disorder (ADHD). In this review, we summarise the developmental profile of the hippocampus during fetal and neonatal life and examine the hippocampal deficits observed following common human pregnancy complications.

**Impact:**

- The review provides a comprehensive summary of the developmental profile of the hippocampus in normal fetal and neonatal life.
- We address a significant knowledge gap in paediatric research by providing a comprehensive summary of the relationship between pregnancy complications and subsequent hippocampal damage, shedding new light on this critical aspect of early neurodevelopment.

## Introduction

The hippocampus lies deep within the medial temporal lobe of the brain and mediates critical functions related to emotional regulation, learning, memory, and cognitive functions. The primary cellular structure and hippocampal form is laid down in utero, ^1^ with postnatal development necessary for the full complement of cellular connections.^2^ Both anatomically and functionally, the hippocampus is a heterogeneous structure, with distinct subfields that differentially regulate learning, memory, and emotions. Common pregnancy complications, which include preterm birth, fetal growth restriction (FGR), intrauterine inflammation, and acute hypoxic-ischaemic insult at birth, can have profound effects on brain development and disrupt the hippocampus with life-long consequences for brain function. The rapid growth of the hippocampus during the third trimester of pregnancy, combined with its high neuronal density, renders it susceptible to injury in the event of intrauterine compromise.^3,4^ Magnetic resonance imaging (MRI) studies confirm that hippocampal structure is altered in human infants in response to perinatal compromise, with reduced hippocampal volume observed in children born preterm or growth restricted.^3,5–8^ A recent meta-analysis demonstrates that preterm-born individuals have smaller hippocampal volume compared to term-born individuals, even after accounting for differences in brain size, indicating that in utero compromise adversely impacts hippocampal growth.^9^ Additionally, pregnancy complications can lead to alterations in the connective pathways between the hippocampus and other brain regions. Subsequent to these structural alterations, short- and long-term functional consequences have been described, including problems in cognition, memory, and motor function.

The breadth of clinical and preclinical studies to examine normal and disrupted hippocampal development has necessitated a two-part review. Part one of this review is focused on normal hippocampal structure and function and provides available evidence from human studies that common perinatal insults disrupt hippocampal development. In the second part of this review^10^, we introduce the preclinical literature which describes the mechanisms underlying altered hippocampal form and function, including impaired neuronal morphology and synaptic connectivity.

### Overview of hippocampal structure

**Fig. 1:**
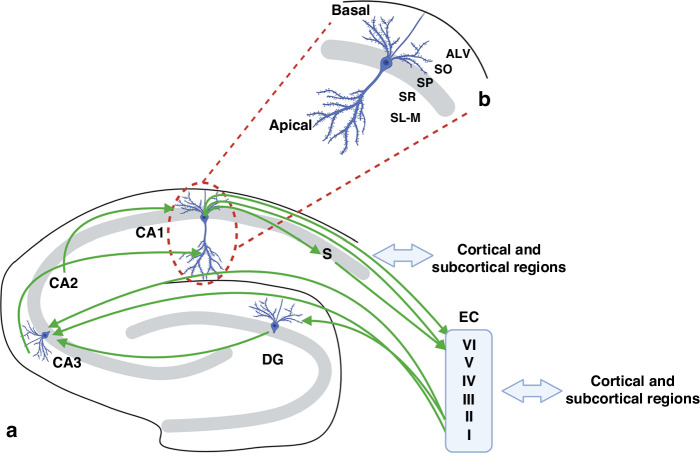
Diagram of the *hippocampal formation*. **a** Diagram depicting connectivity within the hippocampal region, and the connections to the entorhinal cortex (EC) layers (I-VI) and subcortical regions (i.e., thalamus, amygdala, hypothalamus). The distinct hippocampal subfields include the dentate gyrus (DG), Cornu ammonis (CA),1, CA2, CA3, and subiculum (S). **b** The hippocampus is comprised of five layers; stratum alveus (ALV) stratum oriens (SO), stratum pyramidale (SP), stratum radiatum (SR), and stratum lacunosum moleculare (SL-M). Imaged created with BioRender.com (agreement number YW266CRAY9).

*Hippocampus* is derived from the Greek terms for horse (hippo) and sea (kampos), reflecting the resemblance of this structure to a seahorse. It lies within the medial temporal lobe and forms part of the limbic system, regulating emotions, memory, cognitive function, spatial navigation, and motivational processes.^11–13^ The hippocampus comprises four cornu ammonis (CA) subfields (CA1, CA2, CA3 and CA4) and, together with the dentate gyrus (DG), subiculum and entorhinal cortex, is termed the *hippocampal formation*^14–16^ (Fig. 1). These regions of the hippocampal formation form a tightly connected circuit from the entorhinal cortex to the DG, and then into the CA subfields, with outputs from the subiculum to the thalamus, amygdala, hypothalamus, septum, and prefrontal cortex.^15^ The hippocampus is a neuron-rich, five-layer structure; a thin layer of white matter consisting of axons, the stratum alveus (ALV), a pyramidal neuronal layer, stratum pyramidale (SP) with basal dendrites extending to the stratum oriens (SO) and apical dendrites projecting into the stratum radiatum (SR), and stratum lacunosum moleculare (SL-M) layers (Fig. 1b). Supporting the pyramidal cells within the hippocampus are interneurons in the SO and SL-M layers, which are present in many different subtypes, however, all contribute to synaptic connections and cell signalling, and allow the intricacies of the hippocampal circuit to function appropriately.^17–19^

### Hippocampal structure and development

**Fig. 2:**
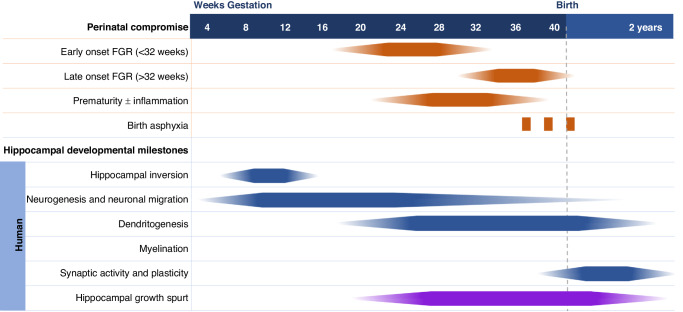
Timeline of perinatal injury in humans relative to hippocampal development milestones. Dark solid colour indicates peak development, or time of insult.

Hippocampal development commences within weeks of conception and continues through the first years of life in human infants (Fig. 2). By week 8−9 of human gestation, the hippocampus is distinguishable from other brain regions, marking the beginning of distinct hippocampal development.^2^ The DG and CA begin as thin structures, and from 10 weeks’ gestation growth rate and thickness increase resulting in the folding of the DG and CA between 13 and 16 weeks’ gestation to form two interlocking C-shaped structures, in a process termed hippocampal inversion.^2,20,21^ Histological assessments from Humphrey^20^ formed the basis for many diagrammatic representations of hippocampal development during gestation, particularly describing this inversion, folding, and sulcation process. From the time of hippocampal inversion until approximately 20 weeks’ gestation represents a period of rapid growth, and it is said that the hippocampus develops faster than most other brain regions during this time, with peak neurogenesis occurring over this period.^1^ By 18 to 21 weeks’ gestation, the cellular foundations of the *hippocampal formation* are in place with the characteristic folded structure and sulcus present, with a near-full complement of pyramidal neurons.^1,21^ Thus, it is said that the hippocampus resembles the ‘adult’ form by mid-gestation in the human.^1,21^ Histological analysis of human fetal and infant hippocampal samples demonstrates that pyramidal neurons are primarily laid down in the first half of pregnancy.^22^

The pyramidal neurons within the hippocampus align in an organised unidirectional formation along the pyramidale layer, residing within the five-layered structure from external to internal hippocampus; ALV, SO, SP, SR, and SL-M,^23^ with the pyramidal neuronal cell bodies sitting within the SP (Fig. 1). Interneurons reside within the SO and SL-M layers and support the connectivity and function of the pyramidal neurons. Formation of this five-layered structure occurs over a prolonged period in late gestation, with the pyramidal neurons following a “climbing” technique from the ventricular zone, where they are generated, to the SP layer, where they will reside. The climbing technique seen in the hippocampus differs from the typical migration of cortical and neocortical neurons^24,25^ and occurs due to the highly branched processes on the migrating hippocampal cells, which make contact with the radial fibres to allow a zig-zag motion through to the crowded SP layer.^25^ In the CA1 region, once the cell bodies of the pyramidal neurons reach their final destination within the SP, the neurons commence neurite outgrowth with basal dendrites extending into the ALV and SO, and apical dendrites projecting down into the SR and SL-M. The migration patterns of neurons within CA3 are thought to be similar to the CA1 neurons,^24,26^ however, less is known about the migration patterns of the other CA regions.

Mature pyramidal neurons have a highly arborised dendritic structure, which is an important determinant in the complexity of functions mediated by the hippocampus.^27^ Dendritogenesis of hippocampal neurons occurs from approximately mid-pregnancy in the human fetus and extends well into infancy.^22^ Structural analysis of CA3 hippocampal neurons in the human brain demonstrated that at 18 weeks’ gestation, both apical and basal dendrites were present but sparsely distributed, while at 33 weeks’ gestation, dendritic arborisation had increased 3-fold and showed a highly developed structure.^28^ Commencing days after a neuron has been generated, neurite sprouting commences, with one neurite extending in length to send the axon to the target area, while remaining neurites grow, extend and branch into the dendritic arbour for the establishment of synaptic connections.^29^ These synaptic connections between cells occur as membranous protrusions along the dendrites called spines. Spines are diverse in shape and length resulting in subtypes (filipodia, thin, stubby, mushroom spines) classification that develop along different timelines; filipodia are long and thin protrusions that exist transiently early in postnatal life and decrease into adulthood.^30^ Thin, stubby and mushroom spines are more stable with long-term potentiation, and regenerate throughout all stages of development, providing strong connections between synapses for optimal hippocampal function.^30^ Bourne and Harris^31^ describe an extensive list of molecular mediators (e.g., PSD-95, CamKII, Actin, N-cadherin) of spine development, stabilisation, and plasticity, highlighting the dynamic and adaptive nature of dendritic spines, which in the hippocampus is likely an important factor for structural and functional plasticity.

In Fig. 2, we broadly describe the developmental profile of the CA1 – CA3 regions. It is crucial, however, to appreciate the important role of the DG as the gateway of the hippocampus. Moreover, there are distinct structural and functional differences between the DG and the CA regions. For example, maturation of the DG occurs later than CA1 – CA3.^32,33^ The DG granule cell layer appears from the 12th week of gestation, with a high rate of cell proliferation from this timepoint through to the 24th week of pregnancy. From the 24th week of gestation, neurogenesis slows significantly but continues to about two years of age, where it then remains lifelong although at a diminished rate.^2,33^ One of the critical differences between the DG and the CA regions lies in the capacity of the DG for ongoing neurogenesis throughout life, with new neurons generated in the subgranular zone (SGZ) of the DG.^34,35^ Thus, the DG is considered to be a unique brain region as it holds a pool of neural stem cells that produce new neurons, contributing to brain plasticity and tissue regeneration.^32^ While knowledge gaps remain regarding the drivers and processes of adult neurogenesis, with likely some overlap between embryonic and adult neurogenesis, it is argued that lifelong neuronal regeneration is confined to the DG.^36^ The synaptic plasticity of DG hippocampal cells is regulated by activity and experiences that result in the formation of new memories and mediate DG neurogenesis.^29^ Interestingly, the granule cells of the DG are more resistant than pyramidal neurons of the CA1 to a number of adverse conditions,^37,38^ and therefore, the majority of the clinical research effort to date investigating hippocampal deficits has focussed on the CA regions.

The vasculature within the CA regions of the hippocampus is relatively sparse given the area of the hippocampus relative to other brain regions,^39^ with fewer, widely spaced microvessels, requiring oxygen to diffuse further into tissue.^39^ The lower vascular density is matched by a relatively low basal blood flow (~50% lower basal blood flow compared to the thalamus or brainstem).^40^ As would be expected, metabolic demand in the hippocampus matches the low vascular density and blood flow, with adjacent hippocampal pyramidal neurons not likely to be active simultaneously, reducing local energy demand compared to cortical regions.^39^ However, the low vascular density but neuron-rich population may explain the susceptibility of the hippocampus to perinatal compromise, as the sparse vasculature is not well suited to rapid adjustments in oxygen supply in response to a hypoxic insult.

### Hippocampal connectivity

The size, anatomical structure, and extensive connectivity within and external to the hippocampus are key to its heterogeneous functionality (Fig. 1). The axons that emanate from neurons in the entorhinal cortex synapse with dendrites of the granule cells of the DG, and axons from granule cells synapse with pyramidal cells in the CA3 region via hippocampal mossy fibres, an important pathway in memory formation.^41^ The hippocampal mossy fibres connect DG granule cells to CA3 pyramidal neurons allowing information to flow in a unidirectional manner to the CA1, and then extend out of the hippocampus proper via CA1 axons.^15^ The DG, therefore, provides a crucial gateway between the entorhinal cortex and the hippocampus proper, with DG neurons receiving the first input and passing information further along the pathway,^41^ with the entorhinal cortex mediating hippocampal communications, acting as the major input and output regulator.^42^ Multiple areas including the amygdaloid complex, medial septal region, and the thalamus, provide extrinsic inputs into the hippocampal circuitry, via the entorhinal cortex, as described by Papex.^43^ Distal to the CA1 region, the subiculum of the *hippocampal formation* is an anatomical transition zone (subiculum means support in Latin), and a major source for hippocampal output into the cortical regions of the brain, thereby directing activity across the brain.^44^

Interrogation of the CA1 pyramidal neuron structure within the hippocampal circuitry reveals the unique roles of the basal and apical dendrites. The apical dendrites receive inputs at various points along the dendrite, from the CA3 neurons via Schaffer collaterals at the proximal end to the soma, and direct glutamatergic input from the entorhinal cortex at the distal dendrites. Conversely, the basal dendrites receive direct inputs from CA2 neurons (Fig. 1).^45–48^ This is an important distinction to consider as there are examples of perinatal compromise that impact only the apical dendrites, impairing both connectivity and functionality of the neuron in a unique manner, compared to an insult that may affect basal dendrites.^49^

Compared to the intra-hippocampal microcircuitry, the extra-hippocampal connections are complex and not well characterised.^50^ In vivo assessment of hippocampal connections undertaken by Maller et al.^50^ revealed six predominant hippocampal pathways – the inferior longitudinal fasciculus, spinal-limbic pathway, anterior commissure, cingulate bundle, fornix and tapetum - all long-range pathways connecting limbic and sub-cortical structures. This connectivity reflects the wide-ranging functionality of the hippocampus and the ability of the hippocampus to moderate multiple brain processes.

The optimal function of the complex internal neuronal network of the hippocampus and the long-range extrinsic connections requires mature myelin. Myelin is the fatty insulation that surrounds the axons and aids the conduction velocity of neurons in the hippocampal pathways.^51^ The developmental profile of myelin within the hippocampus, described by Abraham et al.,^52^ begins at 20 weeks’ gestation with the presence of mature oligodendrocytes and myelinated axons appearing in the hippocampal region between 21-35 weeks. Myelination extends well past birth until adult-like myelin density is present in adolescent tissue,^52,53^ consequent with an increase in hippocampal volume over this period.^54^ It is not yet understood when myelination ceases, however, the increase in hippocampal volume that occurs in childhood is followed by stabilisation or subtle subfield decreases at adolescence, suggesting that adolescence may be the timepoint where myelination is complete.^54^

### Overview of hippocampal function

Functional assessments of the hippocampus have a rich and well-documented history. Famously, the 1953 case of H.M., who lost much of his memory when his hippocampus was removed in an attempt to treat epilepsy, provided the first insight into the primary functions of the hippocampus.^55^ Since then, research has taken great strides to elucidate the function of the hippocampus, including the differential roles of the component sub-regions. The intrinsic circuitry of the hippocampus, as well as the vast connections to cortical and subcortical brain regions, gives rise to multiple functions that span episodic memory, emotional regulation, spatial navigation, learning, and cognition. Further, the distinct structure and connections of the anterior and posterior hippocampus have been shown to underpin separate functional roles, however, this is still to be fully elucidated.^56^ The posterior hippocampus is described as playing a more significant regulatory role in spatial memory as it receives visual and spatial information from the anterior cingulate cortex.^12,56,57^ In contrast, the anterior hippocampus has strong connections with the prefrontal cortex, amygdala, and hypothalamus, favouring emotional processing and autonomic endocrine systems.^12,58^ To date, there is little research that separates the anterior from the posterior hippocampus in the context of hippocampal dysfunction or injury during fetal and neonatal development.

Many functions of the hippocampus, including learning, memory, and spatial navigation, are facilitated by long-term potentiation (LTP),^59–62^ which is the persistent strengthening of synapses that fosters signal transmission between neurons. Long-term potentiation is widely recognised as the cellular mechanism of memory formation.^63^ Within the hippocampus, LTP is shown to regulate hippocampal plasticity with glutamate receptors such as α-amino-3-hydroxy-5-methyl-4-isoxazole propionic acid (AMPA) or N-methyl-D-aspartate (NMDA) integral to this role.^64^ It is these receptors that drive synaptic plasticity, promote LTP, and allow for wide-ranging functionality of the hippocampus. Neuropeptides also play a key role in supporting the neurotransmitters within the hippocampus; somatostatin is one neuropeptide known to significantly contribute to emotion regulation signals.^65^

### The consequences of perinatal insult on the hippocampus

Brain development over the perinatal period is sensitive to disruptions arising from common pregnancy complications, including preterm birth, FGR, and hypoxic-ischaemic encephalopathy (HIE).^66–69^ The strong association between perinatal compromise and structural abnormalities of the hippocampus is evident from clinical studies linking brain imaging outcomes with functional deficits. Key milestones in hippocampal development such as neuronal migration, neurite outgrowth of axons and dendrites, and synaptogenesis are highly active from about mid-pregnancy (20 weeks’ gestation) onwards (Fig. 2), therefore, preterm birth or other complications during pregnancy will significantly disrupt these developmental processes. Further, as the hippocampus is still developing at term, insults occurring around the time of birth, such as perinatal (birth) asphyxia resulting in HIE, can also cause damage.

#### Prematurity

Preterm birth affects approximately 11% of births worldwide and results in significant perturbations in brain development.^70^ Preterm birth can be sub-categorised as extremely preterm (<28 weeks gestation (GA)), very preterm (28–32 weeks GA) and moderate to late preterm (32−37 weeks GA).^71^ There is a multitude of factors and complications that can arise during pregnancy to induce preterm birth including having had a previous premature baby, twin/multiple pregnancy, intrauterine infection, substance abuse, premature rupture of membranes, or impaired development of the baby indicating early delivery.^72^ Numerous studies show that neuropathology associated with the preterm brain is principally via two relatively common upstream insults, hypoxia-ischaemia (HI) and infection/inflammation.^69,73^ Intrauterine inflammation, including placental and amniotic fluid infection (chorioamnionitis), is recognised as a causal factor that both predisposes to preterm birth,^74^ and is also independently associated with altered brain development and brain injury.^75,76^

#### Fetal growth restriction

FGR, also known as intrauterine growth restriction (IUGR), is a common pregnancy complication in which the fetus fails to reach its genetic growth potential in utero.^66^ It affects 6−10% of infants born in high-income countries, and up to six times more infants born in low-income countries.^77,78^ Placental insufficiency is the most common cause of FGR, in which suboptimal placenta structure and function results in reduced transfer of nutrients and oxygen to the developing fetus, thus adversely impacting the trajectory of fetal growth.^79^ FGR is strongly linked to neurodevelopmental deficits across the domains of cognition and learning, motor function, and behaviour.^66,80–83^ Previously, the term small for gestational age (SGA) was also used as a proxy to describe FGR, but SGA generally includes all infants who were below the 10th percentile for weight and therefore does not necessarily include evidence of placental insufficiency.^84^ The 2016 consensus definition of true FGR includes infants with an estimated fetal weight <10th percentile for gestation and sex together with Doppler indices of disrupted uteroplacental blood flow, or estimated fetal weight <3rd percentile as a sole parameter.^84^ FGR can be further classified as early-onset FGR, diagnosed before 32 weeks’ gestation, or late-onset, diagnosed after 32 weeks. Early-onset FGR appears to portend worse neurological outcomes than late-onset FGR.^66^

#### Hypoxic-ischaemic encephalopathy

HIE is a condition of disrupted neurological function as a consequence of severe or prolonged hypoxia-ischaemia at the time of birth.^85^ HIE is a devastating condition, related to the death of 1 million infants in their first month of life, while 25% of children who survive will have long-term debilitating conditions such as cerebral palsy.^86,87^ An acute hypoxic-ischaemic insult and subsequent HIE can affect infants born prematurely or at term, and therefore the consequences of HIE are broad.^88^ Most commonly, HIE is linked to an acute, severe, asphyxic event at birth, which induces a well-described injury cascade with distinct phases of metabolic disturbance and injury that ultimately result in the degeneration of neurons,^89^ and this progression of injury is best described in infants born at term.^73^ Placental abruption, uterine rupture, or umbilical cord compression are common causes of an acute hypoxic insult that may lead to HIE.^85^

These three perinatal conditions have been highlighted in this review as they are prominent pregnancy complications and have profound impacts on hippocampal development, morphology, and function. The clinical studies detailed below describe the consequences of each of these conditions on the hippocampus, however, it is important to note that due to the intricate nature, size, and location of this brain region, imaging techniques are limited, thus accompanying preclinical investigations are needed to dissect mechanisms of disease^10^.

### Impact of perinatal insults on hippocampal structure

A large number of clinical studies have investigated the impacts of preterm birth, very low birth weight (VLBW), FGR, and HIE on hippocampal morphology and function (Table 1). The studies summarised in Table 1 have been selected as they examined the impact of these insults on hippocampal structure and their association with functional outcomes. Collectively, these studies demonstrate that prematurity ( ± inflammation), VLBW, FGR, and HIE, are all clearly associated with a total reduction in hippocampal volume measured using MRI imaging techniques such as T1-weighted and T2-weighted imaging, segmentation, and hippocampal shape analysis.^3,5–8,74,90–112^ As one example, MRI voxel-based morphology was used to detect significant volume loss in both the left and right hippocampus of adolescents with a history of prematurity.^94^ Further to reductions in hippocampal volume, Lammertink et al.^95^ utilised MRI data of preterm infants and found a reprioritisation of neurodevelopmental trajectory to the amygdala and insula in response to preterm birth (mean 26 weeks GA), resulting in a reduced volume of connections in the hippocampus, parahippocampal gyrus and fusiform area measured by constructed maturational covariance networks relative to grey matter volumes.^95^ Another study of premature-born adults assessed at 20 years of age, used functional MRI (fMRI) to investigate the impact of prematurity and found reduced fractional anisotropy tracts passing through the thalamic and hippocampal regions of the preterm group, resulting in altered activation patterns of the hippocampus during retrieval tasks.^96^ This study highlights the impact of prematurity on impaired connectivity of key structures of the learning and memory network, including the anterior cingulate and caudate body, thalamus, and hippocampus and consequently, hippocampal function.^96^

**Table 1:** The impact of perinatal compromise in human studies.

Pregnancy complication	Participants	Sample size	Hippocampal morphology	Hippocampal function	Reference
Prematurity	Preterm infants: <36 weeks GA.	71 participants	↓ total hippocampal volume.	NA	Ball et al.^90^
Prematurity	Extremely preterm infants <28 weeks GA vs. term born controls, assessed at 18 years of age.	Extremely preterm; *n* = 148 Control; *n* = 132	↓ 8% hippocampal volume in extremely preterm.	Smaller brain volumes associated with lower IQ and poorer educational skills.	Cheong et al.^91^
Prematurity	Preterm infants (<33 weeks GA) were assessed at 15 years and 19 years of age.	Preterm; *n* = 61 Control; *n* = 35	Significant surface deformations reflecting atrophy of the hippocampus.	↑ right hippocampal volume and bilateral anterior surface deformations were associated with delusional ideation scores.	Cole et al.^92^
Prematurity	Preterm infants (<32 weeks GA) vs. term-born controls (~40 weeks GA).	Preterm; *n* = 53 Control; *n* = 361	↓ 4% volume in the right hippocampi of preterm infants.	NA	Ge et al.^93^
Prematurity	Preterm infants (25-35 weeks GA), assessed at age 10 to 18 years.	22 participants	↓ total hippocampal volume, with left posterior dominance.	Associations between left hippocampal grey matter reductions and verbal memory.	Gimenez et al.^94^
Prematurity	Extremely preterm infants: <28 weeks GA, admitted to the NICU and exposed to “early-life stress”.	Low stress preterm; *n* = 90 High stress preterm; *n* = 90	Infants exposed to high stress showed lower covariance within the default mode network, rendering them at higher risk for stress-related psychopathology.	NA	Lammertink et al.^95^
Prematurity	Preterm infants (<33 weeks GA), assessed at 20 years of age	Preterm; *n* = 21 Control; *n* = 10	↓ fractional anisotropy tracts in the hippocampal region of VPT.	Different pattern of activation during retrieval/recall in hippocampus of VPT.	Salvan et al.^96^
Prematurity	VPT infants (<32 weeks GA), assessed at 2 years of age.	VPT; *n* = 85 Brain injury; *n* = 73 Control; *n* = 55	↓ 7% hippocampal volumes in very preterm infants.	Smaller hippocampal volumes were related to worse motor performance at age 2.	Strahle et al.^97^
Prematurity with and without GM-IVH	Preterm birth <37 weeks GA, having a neonatal diagnosis of GM-IVH assessed at age 6 to 16 years old.	Preterm w/GM-IVH; *n* = 16 Preterm; *n* = 20 Control; *n* = 22	↓ left and right global hippocampus volume in high-risk preterm sample with GM-IVH compared to both low-risk preterm and full-term groups.	FIQ was within normal limits in both preterm groups, it was significantly lower compared to full-term children.	Fernandez de Gamarra-Oca et al.^98^
Prematurity and chorioamnionitis	Preterm birth between 28 and 37 weeks GA, with and without chorioamnionitis, assessed at 8-9 years old.	Preterm w/chorioamnionitis; *n* = 11 Preterm; *n* = 16	↓ right hippocampal volume in children exposed to chorioamnionitis.	NA	Hatfield et al.^74^
Prematurity, VLBW, and perinatal brain injury	VPT (<32 weeks GA) and perinatal brain injury vs. VPT no injury vs. control.	VPT w/brain injury; *n* = 16 VPT no injury; *n* = 13 Control; *n* = 14	↓ 5% total hippocampal volume in very preterm group ↓ 14% total hippocampal volume perinatal brain injury group.	NA	Froudist-Walsh et al.^7^
Prematurity/VLBW	Preterm infants: <30 weeks GA or BW < 1250 g.	227 participants	↓ total hippocampal volume.	Children with working memory deficits had marginally smaller hippocampi.	Beauchamp et al.^3^
Prematurity/VLBW	VPT infants born <30 weeks GA or BW < 1250 g, assessed at 13 years	VPT w/anxiety; *n* = 16 VPT no anxiety; *n* = 108	VPT children who met anxiety disorder criteria showed slower hippocampal growth trajectories	Impaired hippocampal development associated with anxiety disorder	Gilchrist et al.^99^
Prematurity/VLBW	Premature born (< 32 weeks GA and/or BW < 1500 g), assessed at 26 years of age.	Preterm/VLBW; *n* = 103 Control; *n* = 109	↓ hippocampal subfields volumes.	Correlations between all left-sided functional hippocampus units and adult FIQ	Hedderich et al.^5^
Prematurity/VLBW	VPT infants either <30 weeks GA or a BW of <1250 g, assessment at 2, 5 and 7 years of age.	VPT; *n* = 145 Control; *n* = 34	↓ 5% right and ↓ 6% left hippocampal volumes in VPT children.	Neither left nor right hippocampal volumes were associated with performance on memory or learning outcomes.	Omizzolo et al.^100^
Prematurity/VLBW	VPT infants either <30 weeks GA or a BW of <1250 g, MRI at term equivalent age, assessed at 5 years old	165 participants	↓ right and left hippocampal volumes	In females, smaller hippocampal volumes were associated with hyperactivity, inattention and peer problems.	Rogers et al.^101^
Prematurity/VLBW	Preterm infants: <30 weeks GA or BW < 1250 g vs. full-term infants: >37 weeks GA.	Preterm; *n* = 184 Control; *n* = 32	↓ 3% hippocampal volumes in preterm group.	Infants with reduced corrected hippocampal volume at 2 years showed reduced MDI scores.	Thompson et al.^6^
Prematurity/VLBW	VPT infants: <30 weeks GA or BW < 1250 g vs. full-term infants: >37 weeks GA, assessed at 7 years of age	VPT; *n* = 184 Control; *n* = 32	VPT children showed less hippocampal infolding ↓ growth between infancy and 7 years in VPT	Hippocampal developmental trajectory did not predict learning and memory deficits.	Thompson et al.^102^
VLBW	Infants born with BW < 1500 g, vs. control, assessed at 19 years of age.	VLBW; *n* = 44 Control; *n* = 61	↓ 5% total intracranial volume. ↓ 7% absolute hippocampal volume.	Inferior memory function correlated to the volume of hippocampi, BW, and perinatal morbidity.	Aanes et al.^103^
VLBW	School aged children (6−11 years) born preterm <34 weeks GA and <1500 g, vs. control	VLBW; *n* = 34 Control; *n* = 104	↓ 3% left hippocampal volume. ↓ hippocampal subfield volume.	↓ scores on spatial span and digit span assessments, for verbal and visual working memory.	Aanes et al.^104^
VLBW	VLBW: <30 weeks GA and <1500 g vs. control: median GA 39.5 weeks, median BW 3622 g.	VLBW; *n* = 11 Control; *n* = 8	↓ 15% hippocampal volumes in VLBW children compared with age-matched, full-term controls.	VLBW children had deficits in everyday memory.	Isaacs et al.^8^
SGA	SGA: BW and/or birth length -2 SD of normal vs. control, assessed at 4−7 years old.	SGA; *n* = 34 Control; *n* = 18	SGA children showed less activation in the left parahippocampal region compared to control.	SGA children had lower IQ scores than control children and had slower performance in encoding and recognition tasks	De Bie et al.^105^
FGR	Premature infants (32 weeks GA) born with FGR (<3rd centile) vs. premature control infants.	FGR; *n* = 13 Control; *n* = 13	↓ GM volume of both hippocampi and ↓ 9% total hippocampal volume.	↓ APIB scores in FGR infants. ↓ mean MDI in FGR infants.	Lodygensky et al.^106^
FGR	Premature infants (<34 weeks GA) with FGR (<3rd centile) vs. premature infants with appropriate BW vs. control, assessed at 12 months old.	Preterm FGR; *n* = 18 Preterm; *n* = 15 Control; *n* = 15	↓ WM in hippocampus of FGR infants vs. control.	↓ scores on motor, fine motor and adaptive behaviour on the BSITD.	Padilla et al.^107^
FGR	Preterm infants (<33 weeks GA), born FGR (<3rd centile) vs. AGA. MRI at term equivalent age, assessment at 22 months age.	VPT FGR; *n* = 49 VPT Control; *n* = 265	↓ volume of GM in limbic brain regions (including the hippocampus).	↓cognitive and motor outcomes. ↑ autism screening risk at 22 months.	Sacchi et al.^108^
Perinatal hypoxia-ischaemia	Infant brain cases taken from autopsies, six diagnosed as hypoxic.	Hypoxia; *n* = 6 Control; *n* = 4	↓ somatostatin and neuropeptide expression in pyramidal cell layer and stratum oriens of CA1.	NA	González Fuentes et al.^109^
Perinatal hypoxia- ischaemia	Children born >36 weeks GA, acute perinatal asphyxia, with and without therapeutic hypothermia.	HIE w/hypothermia; *n* = 15 HIE; *n* = 17	Atrophy of the hippocampal and parahippocampal white matter with HIE.	Neurocognitive and memory problems at 10 years of age.	Annink et al.^110^
Perinatal hypoxia-ischaemia	Term-born children with mild and moderate HIE.	Mild HIE; *n* = 26 Moderate HIE; *n* = 26 Control; *n* = 37	↓ hippocampal volumes with moderate HIE.	↓ hippocampal volumes were associated with poorer long-term visuospatial memory.	Annink et al.^111^
Hypoxic ischaemic encephalopathy	Term-born children with neonatal HIE, treated with hypothermia	HIE; *n* = 10 Controls; *n* = 8	↓ hippocampal volumes with HIE.	Within-group correlation between the hippocampal volume and memory scores in children with HIE.	Pfister et al.^112^

*APIB* assessment of preterm infants’ behaviour, *BSITD* Bayley scale for infant and toddler development, *BW* birth weight, *FIQ* full intelligence quotient, *GA* gestational age, *GM* grey matter, *GM-IVH* germinal matrix-intraventricular haemorrhage, *HIE* hypoxic-ischaemic encephalopathy, *IQ* intelligence quotient, *MDI* mental development index, *MRI* magnetic resonance imaging, *NA* not assessed, *VLBW* very low birth weight, *VPT* very preterm, *SGA* small for gestational age, *w*/ with, *WM* white matter.

Relative to prematurity, less is known about the impact of FGR on hippocampal form and function. Studies that have investigated infants classified as FGR or VLBW often include co-morbidity with prematurity in their study cohort, as naturally many FGR or VLBW infants are born prematurely. Thus, it is often difficult to disentangle the effect of prematurity and growth restriction. Despite this, FGR has consistently been associated with a reduction in hippocampal volume.^8,104,106–108,113^ In infants born preterm, (<32−34 weeks GA), MRI voxel-based morphometry showed that in growth restricted preterm infants, hippocampal grey matter volume^106^ and hippocampal white matter volume were reduced.^107^ Further, one study of SGA infants born at term provides unique insight into the impact of reduced birth weight with the absence of prematurity and found with fMRI analysis less activation in the left parahippocampal region compared to control children.^105^

Due to the nature of studying neurodevelopmental conditions in humans, assessing hippocampal structure beyond volumetric analysis is challenging. One study, by Gonzalez Fuentes et al. ^109^ studied the brains of six infants who had been diagnosed with hypoxic exposure, and at autopsy found a reduction in somatostatin and neuropeptide expression in the pyramidal cell layer and stratum oriens of the CA1 region. The reduction of key neurotransmitters in this autopsy study suggests that the hippocampus would likely have impaired function.^109^

Collectively, these studies demonstrate that the hippocampus is particularly vulnerable to perinatal compromise, with volume deficits a common consequence of a broad spectrum of pregnancy and birth complications. The reduction in hippocampal volume may be indicative of neuronal loss or lack of synaptic arborisation, impairing its intricate connectivity, with implications for hippocampal function. To further reveal mechanisms driving hippocampal volume loss seen with prematurity, VLBW, FGR and HIE, preclinical studies are essential. However, all conditions disrupt the intricate development profile of this brain region across gestation.

### Impact of perinatal insults on hippocampal function

Many studies of perinatal compromise utilise MRI to assess hippocampal volume and morphology, often pairing this structural analysis with various neurocognitive assessments to reveal any deficits in function. However, this field is currently lacking studies that have utilised objective functional assessment such as fMRI to reveal hippocampal-specific impairments (Table 1).^96,105^ Nonetheless, the current literature shows pervasive functional deficits associated with prematurity, VLBW, FGR and HIE, as evidenced by a number of learning, memory, and cognitive assessments.

Follow-up studies of children and adults who were born preterm have investigated the persistent functional impacts of perinatal compromise and a potential relationship to altered hippocampal development. As shown by Gimenez and colleagues,^94^ where a significant correlation between deficits in left hippocampal volume and memory dysfunction in children born prematurely was found, and authors noted “the lower the volume, the lower the level of learning”, emphasising the structure-function relationship.^94^ Cole et al. ^92^ investigated the psychiatric outcomes of adolescents born very preterm compared to adolescents born at term. Using the Peters Delusion Inventory examination, they showed that delusional ideation scores were associated with anterior surface deformation of the hippocampus, thus linking long-term neurodevelopmental consequences of preterm birth with hippocampal structure.^92^ Fernandez de Gamarra-Oca and colleagues^98^ found that while adolescents born preterm (mean 28 weeks gestation) had Full Intelligence Quotient (FIQ) scores within a normal range, the FIQs were significantly lower than term-born adolescents.^98^ Another study undertaken in adults found that those who were born preterm had a persistent reduction in hippocampal volume that correlated with reduced FIQ scores.^5^ In school-age preterm-born children, reduced hippocampal subfield volumes were associated with impaired working memory function.^104^ Further, ex-preterm adults presented with strikingly different patterns of activation in memory recall tasks, indicating that connections between the hippocampus and other brain regions were impaired.^96^ To assess functional outcomes of preterm children, Thompson, et al. ^6^ used the Mental Development Index (MDI) of the Bayley Scales of Infant Development to show that infants with reduced corrected hippocampal volume at 2 years of age showed reduced MDI scores, indicative of reduced cognition.

The posterior hippocampus is fundamental to memory formation and retrieval, particularly spatial memory.^56^ Memory deficits are observed in preterm and VLBW children with hippocampal volume loss.^3,103^ Typically, motor function is not assumed to be directly related to the hippocampus, however, a study by Strahle et al. ^97^ found that reduced hippocampal volumes in children born preterm were related to worse motor performance. Further, it has been shown that the volume of hippocampal subfields CA2 and CA3 is associated with motor sequence learning and memory.^114^ A critical detail missing from research examining the impact of perinatal compromise on the hippocampus is that most studies do not specify whether volumetric differences were observed in the anterior hippocampus, posterior hippocampus, or both. The lack of specificity in reporting results hinders our ability to gain a comprehensive understanding of the structure-to-function relationship between these hippocampal regions, and whether differential vulnerability to injury exists.

As discussed above, a challenge exists in our understanding of the impact of FGR on hippocampal function, as most clinical studies are confounded with prematurity. For example, one study of growth-restricted preterm infants revealed both less mature scores on the Assessment of Preterm Infants’ Behaviour and reduced hippocampal volume at term-equivalent age compared to control infants.^106^ At the 2-year follow-up, neurocognitive dysfunctions persisted and were correlated with a reduction in hippocampal volume.^106^ The study of SGA term-born infants by De Bie et al. ^105^ used fMRI, and found less activation in the left parahippocampal region in SGA children compared to controls, with SGA children demonstrating lower IQ scores and slower performance in encoding and recognition tasks.^105^ There is a paucity of studies investigating late-onset FGR and hippocampal function/development. As such, it is not possible to understand the potential distinction between the impact of early-onset and late-onset FGR in studies on hippocampal development and subsequent function. Whilst not investigating hippocampal structure specifically, two studies by Geva et al.,^115,116^ assessed children diagnosed at birth with FGR and found lower IQ, memory impairments, and more frequent neuropsychological difficulties including executive functioning, inflexibility-creativity, and language. Future research should aim to improve our understanding of these observed functional impairments with accompanying analysis of hippocampal structure, to uncover the depth of this association, particularly in late-onset FGR.

When investigating children who had been diagnosed with moderate HIE as infants, it was shown that working memory, processing speed, and motor outcomes were significantly reduced compared to children with mild HIE.^111^ Further, at 10 years of age, neurocognitive and memory problems were persistent in children born with HIE.^110^

Knowledge of neurodevelopmental disorders that affect language, skills, behaviour, social interactions, and attention, such as Autism Spectrum Disorder (ASD) and Attention-Deficit/Hyperactivity Disorder (ADHD), is rapidly expanding. The aetiology associated with these disorders is complex, with no singular causal pathway underlying their manifestation, but rather a combination of genetic and environmental factors, including conditions of perinatal compromise. MRI studies find that both ASD and ADHD are associated with reductions in hippocampal volume.^117^ It is also postulated that disruptions in the connectivity between the hippocampus, amygdala, and orbitofrontal cortex may contribute to common hallmarks of ADHD, including behavioural disinhibition.^108,118^ To date, work to reveal these associations between perinatal compromise, disrupted hippocampal development, and neurodevelopmental disorders is limited, however, it has been flagged as an area that requires further investigation.^119^

## Conclusions

The clinical evidence presented within this review, focused on normal hippocampal development, followed by pregnancy and birth complications, clearly indicates that the hippocampus is highly susceptible to perinatal compromise. Premature birth FGR and HIE are common complications of pregnancy, and all have the potential to impact the gross structure and organisation of the hippocampus, with negative consequences for long-term function. The most frequent pathological observation in clinical studies of perinatal compromise and hippocampal development is a reduction in total hippocampal volume. Reduced hippocampal volume is seen across all perinatal complications outlined in the current review, as evidenced from the MRI undertaken from as early as term-equivalent age in infants born preterm^90^ through to adolescence.^5^ Current literature has established an association between suboptimal hippocampal structure and deficits in learning and memory. Moreover, damage to the hippocampus has profound and lasting impacts on behaviour and motor function and is associated with a range of neurodevelopmental disorders. In Part 2 of this review^10^, we focus on the extensive preclinical literature on this topic which gives insight into mechanisms underlying observed hippocampal deficits, and potential therapeutic targets designed to protect the hippocampus in the presence of pregnancy complications.
